# Evaluating the effectiveness of *Pisolithus tinctorius* in enhancing the *Eucalyptus*’ resistance to salt stress

**DOI:** 10.1186/s13568-024-01799-w

**Published:** 2025-01-04

**Authors:** Mona S. Zayed, Aya G. A. Ahmed, Shawky M. Selim, Dalia A. Abd El-Fattah

**Affiliations:** 1 Department of Agricultural Microbiology, Faculty of Agriculture, Ain Shams University, Cairo, Egypt; 2 Central Laboratory for Agricultural Climate, Agricultural Research Center, Dokki, Giza, Egypt; 3 Climate Change Information Center, Agricultural Research Center, Giza, Egypt

**Keywords:** *Pisolithus tinctorius*, *Eucalyptus globulus*, Molecular identification, In vitro mycorrhization, Salt stress

## Abstract

Afforestation projects on saline land, using *Eucalyptus* trees and ectomycorrhizal fungi, are crucial for restoring affected areas and promoting ecological and economic benefits, particularly in saline-affected areas. This study was conducted to isolate *Pisolithus* sp. and estimate its potential to improve the growth performance of *Eucalyptus globulus* seedlings under salt-stress conditions. *Pisolithus* sp. was isolated from mature sporocarps and identified through 18S rDNA. *Pisolithus* sp. was evaluated for its response to varying pH values, temperatures, and salinity levels. A pot experiment was conducted to assess the *Pisolithus* strain’s effectiveness in reducing soil salinity’s impact on the growth of *Eucalyptus globulus* seedlings in sandy soil. The identified *Pisolithus tinctorius* with an accession number of OM125275 revealed the highest mycelium dry weight of 0.09 g/100 ml medium at pH 5.8, 0.08 g/100 ml medium at 28 °C, and 0.12 g/100 ml medium at 4% NaCl. *Eucalyptus globulus* seedlings inoculated with *Pisolithus tinctorius* demonstrated significant improvement in most parameters compared to non-mycorrhizal (control) seedlings under salt stress. The seedlings inoculated with *Pisolithus tinctorius* and irrigated with 6 dS/m saline water revealed the highest shoot height (55.670 cm), root length (42.33 cm), shoot fresh weight (6.44 g/plant), root fresh weight (1.84 g/plant), shoot dry weight (2.37 g/plant), and root dry weight (0.810 g/plant) when compared to all treatments. Our findings suggest that selecting appropriate fungal strains is crucial for improving plant performance in saline conditions.

**Graphical abstract:**

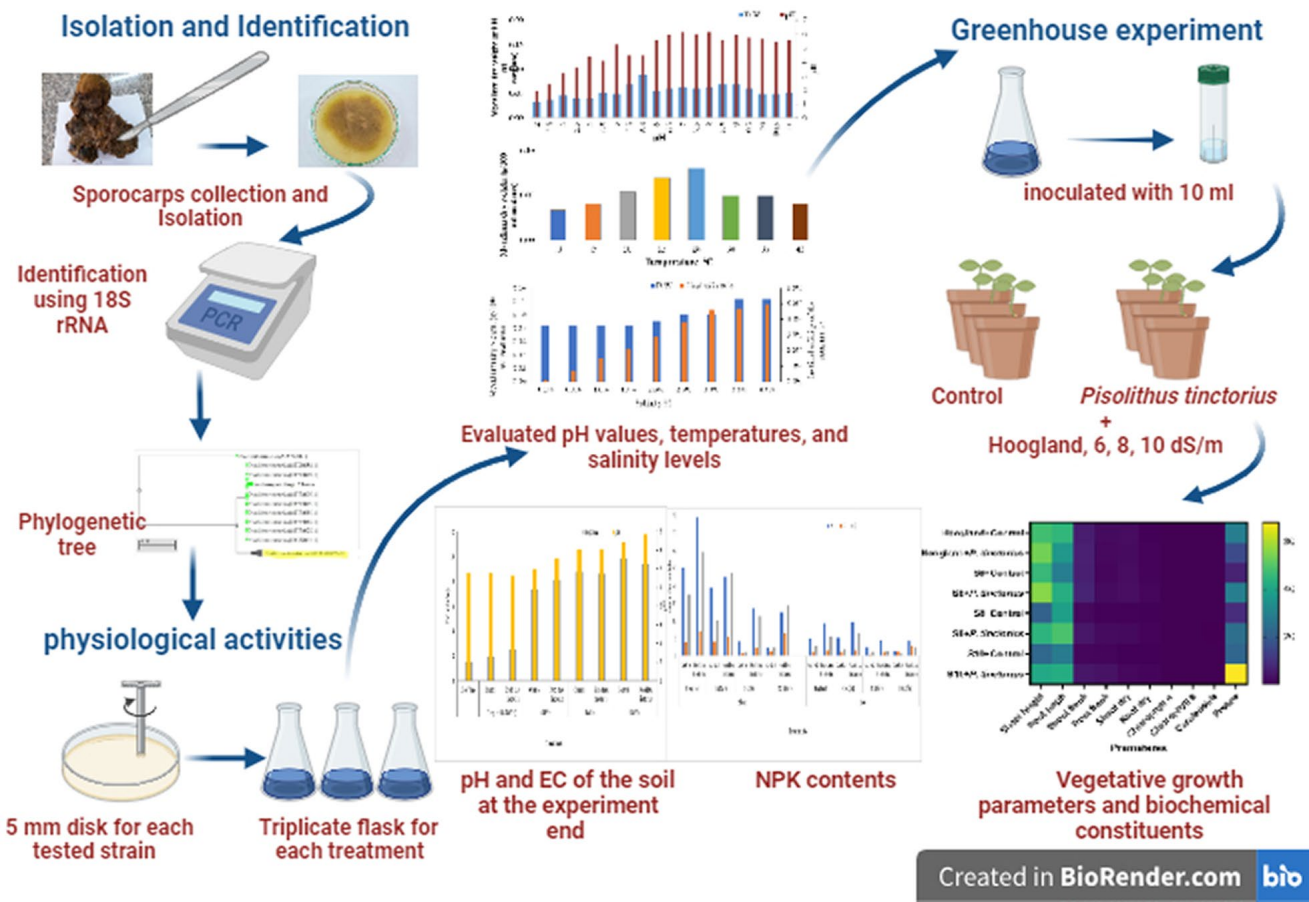

**Supplementary Information:**

The online version contains supplementary material available at 10.1186/s13568-024-01799-w.

## Introduction

Ectomycorrhizae (ECM) is a mutualistic relationship between soil fungi and plant roots, mostly woody trees (Clasen et al. 2018). This association consists of a fungal-mycelium system attaching to plant roots (Brundrett and Tedersoo 2018; Shrestha et al. 2021). In this relationship, the fungal partner supplies nutrients to plants in exchange for the carbohydrates produced during photosynthesis (Xie et al. 2021). Most ectomycorrhizal fungal species belong to Basidiomycotina, Ascomycotina, and some Zygomycotina (Bonfante and Venice 2020; Bai et al. 2021a, b).

The hyphae of ECM fungi entangle on the plant roots, forming a fungal mantle and penetrating between the root epidermis and cortical cells in a so-called Hartig net, which is known as a place of enormous two-directional exchanges of nutrients between the fungus and its host plant. Finally, the external extraradical mycelium emerges from plant roots and extends into the soil for a long distance (Zayed et al 2007; Clasen et al. 2018; Xie et al. 2021).

Ectomycorrhizal symbiotic associations are crucial for plant health and growth as they improve plant tolerance or resistance to abiotic stressors such as drought, high temperature, elevated pH levels, and salinity (Otgonsuren et al. 2016). Furthermore, ectomycorrhizal fungi enhance the provision of water and nutrients to plants, generate enzymes and various organic acids, shield plants from soil pathogens, and enhance soil quality (Prieto et al. 2016).

While abiotic stressors have been shown to affect ECM diversity (Wang et al. 2017; Kumar and Atri 2018), other studies have provided clear proof that certain ectomycorrhizal fungi can protect plants from damage and enhance their resistance to a range of abiotic stressors. For example, *Pisolithus* sp. in drought stress prevents plant damage, whereas *Pisolithus tinctorius* in pecan seedlings (*Carya illinoensis*) allows it to tolerate acidic soil (Kumar and Atri 2018). While Scleroderma bermudense improves mineral nutrition (N and P), decreases sodium ion (Na +) uptake, and increases proline concentration in leaves under salinity stress (Otgonsuren et al. 2016).

*Eucalyptus globulus* is a tall evergreen tree that belongs to the Myrtaceae family, cultivated in most sub-tropical and temperate regions, and has been reported to host a wide diversity of ECM fungi (Dezsi et al. 2015; Surbhi et al. 2023). It is rich in phytochemicals and the essential oils extracted from its leaves and fruits, which have antibacterial, antidiabetic, antitumor, antioxidant, and antifungal properties. In the food industry, their oil is used in the preparation of chewing gums, candies, and antimicrobial packaging. It is also used in aromatherapy as a cure for colds and coughs as well as a stress relaxant (Surbhi et al. 2023). Lastly, *Eucalyptus* sp. is crucial for sourcing raw materials, including wood chips, pulp, energy, fuel, piling, poles, and fiberboards used in furniture and construction.

Using *Eucalyptus* sp. inoculated with ECM fungi for afforesting saline lands is an essential ecological approach that enhances ecosystem restoration and biodiversity conservation. These ECM symbioses enable fungi to mineralize organic compounds through organic acid production, making essential minerals available for plant uptake (Shah et al. 2016; Santana et al 2020). Additionally, ECM seedlings protect plants against pathogens by altering the rhizosphere environment, rendering it less favorable for root pathogens (Santana et al. 2020). The deep roots of *Eucalyptus* sp., in the presence of EM fungi, can reclaim saline lands for productive purposes, lower the saltwater table (Leksungnoen and Andriyas 2019), as well as reduce deforestation.

This study aimed to isolate and identify *Pisolithus* sp., evaluate its tolerance to various abiotic stresses in vitro, and estimate its potential to mitigate the impact of salt stress on the growth of *Eucalyptus globulus* seedlings in sandy soil.

## Materials and methods

### Sporocarps collection and characterization

Young/ mature and undamaged *Pisolithus* sporocarps were collected from clay land near *Eucalyptus* spp. trees located in Al-Azhar University, Cairo, Egypt, during the spring season (March), to be used for the isolation of ectomycorrhizal fungus. The sporocarps were deposited into waxed paper bags and kept in a cool box during transfer to the laboratory. The anatomic-morphological characteristics of the sporocarps were described according to the methods depicted by Brundrett et al. (1996); Agerer and Rambold (2004).

### Isolation of ectomycorrhizal fungi.

The sporocarps were brushed to remove the adhering soil particles and then carefully opened (Brundrett et al. 1996; Obase et al. 2009; Repáč 2011). Small pieces of tissue (2 mm^3^) were removed with fine forceps and placed on a modified Melin-Norkrans (MMN) agar medium (Marx 1969) in Petri dishes. Next, the plates were incubated at 25–28 °C and observed under a stereomicroscope for the initial fungal growth. Finally, the pure cultures were maintained in solid MMN at 25 ± 1 °C and routinely subcultured into a fresh medium every 2 months according to the method described by Ahmed et al. (2022).

### Molecular identification of *Pisolithus* isolates

The *Pisolithus* isolate was identified at the molecular level using the universal 18S rDNA primers ITS1 (ITS1, 5′-TCCGTAGGTGAACCTGCGG-3′) according to the method described by Amicucci et al. (1998).

### Ectomycorrhizal synthesis in sterile culture

Healthy and uniformly sized seeds of *Eucalyptus* sp. were surface sterilized using 5% sodium hypochlorite (NaOCl) for 20 min. The sterilized seeds were rinsed carefully (3–5 times) with sterile water before being individually placed on the Petri dishes (15 cm diameter) containing MMN medium, solidified with 2% agar to induce seed germination. Hyphal plugs 5 mm in diameter were taken from the edge of twenty-day-old colonies of *Pisolithus* isolate. These discs were placed approximately 1.5 cm from the center of the plates, then the Petri dish’s edges were sealed with parafilm and incubated at 28 °C for approximately 2 weeks in the dark until sufficient hyphal growth occurred. The germinated seeds with short radicle roots were placed axenically in a row 3 cm above the level of the outer growing hyphae in the plates inoculated with fungal discs. The plates were incubated in a controlled chamber with a temperature and light level that was adequate for seedling growth (Brundrett et al. 1996). At the end of the experiment (30 days), seedlings’ roots were collected for a photograph with the semithin method.

### Semithin sections of ectomycorrhizal root

Ectomycorrhizal root samples were fixed in 3% glutaraldehyde for 5 min at room temperature, then soaked in phosphate buffer and post-fixed in a potassium permanganate solution. Dehydration involved a 15-min ethanol series (10–90%) followed by 30 min in concentrated ethanol. Samples were transitioned through epoxy resin and acetone before immersion in pure resin. Blocks were trimmed to create trapezoid-shaped faces under a stereomicroscope, less than 1 mm wide and high, to maximize root material visibility. The surface resin was removed using an ultramicrotome (Leica Ultracut UCT) and glass knives. Semithin slices were stained with Toluidine blue and observed under a light microscope (Brundrett et al. 1996; Yaseen and Amin 2021).

### *Pisolithus* sp. preparation for the assessment of some physiological activities

*Pisolithus* sp. growing on MMN solid medium was incubated at 28 °C for 20 days and used to inoculate the sterilized flasks containing 100 ml of MMN medium within a 5 mm disk from the edge of each tested strain.

### Effects of different pH values on the growth of ***Pisolithus*** sp.

The MMN liquid medium was adjusted to various pH levels ranging from 2 to 11 ± 0.5 by adding 1N HCl or 1N NaOH. The sterilized medium was inoculated with a 5 mm disk of *Pisolithus* strain and incubated at 28 °C for 24 days. Triplicate flasks were used for each treatment. The mycelium dry weight (g/100 ml medium) was used to measure the fungal growth at the end of the incubation time, and the final pH of the culture medium for each treatment was noted.

### Influence of different temperatures on the growth of ***Pisolithus*** sp.

A five-mm disk of the *Pisolithus* strain was used to inoculate the MMN liquid medium. The medium was then incubated at temperatures ranging from 15 to 30 °C for 24 days, with three replicates per treatment. At the end of the incubation period, the growth of the tested strain was estimated (g/100 ml medium).

### Influence of different salinity stress levels on the growth of ***Pisolithus*** sp.

MMN liquid medium containing different NaCl percentages, ranging from 0.1 to 4.0% NaCl, was inoculated with a 5 mm disk of *Pisolithus* strain and incubated at 28 °C for 24 days in triplicate flasks for each treatment. At the end of the incubation period, the growth of the tested strain was estimated (g/100 ml medium). The EC of the medium broth was measured at the end of the experiment using an EC meter (Portable High Range EC/TDS Meter-HI99301- Hanna Instruments- USA).

#### Pot experiment

A pot experiment was conducted using a completely randomized design with five replicates per treatment at the greenhouse of the Microbial Inoculants Center, Faculty of Agriculture, Ain Shams University, Cairo, Egypt, to assess the effectiveness of the *Pisolithus* strain in reducing the influence of salinity stress on the growth performance of *Eucalyptus globulus* seedlings in sandy soil.

*Eucalyptus globulus* seedlings were cultivated in polyethylene bags (15 × 30 cm in diameter) filled with 4 kg of sandy soil. Each seedling was inoculated with 10 ml of the *Pisolithus* strain three consecutive times (7, 15, and 30 days after planting). The seedlings were irrigated with saline water containing varying levels of sodium chloride (NaCl) at 6, 8, and 10 dSm^−1^. The experimental layout was completely randomized design with those replications.

#### Assessment the growth parameters of seedlings

At the end of the six-month growing period, three seedlings were randomly selected and harvested carefully from each treatment, and their growth parameters were estimated, including plant fresh and dry weights (g/plant), shoot, and root length (cm/plant).

#### Determination of some biochemical constituents in the seedlings

Total N, P, and K% were estimated in seedlings’ shoots and roots in accordance with the method stated by Jackson (1973) at the end of the experiment.

The chlorophylls were determined according to the method described by Lichtenthaler and Wellburn (1983) and modified by Porra (2002). Chlorophyll a, b, and β-carotene were measured using a UV-Spectrophotometer (SPAD-502Plus) at wavelengths 645, 663, and 470 nm, respectively. Chlorophyll and carotenoid contents were calculated as follows:

$$\mu {\text{g Chl }}a/g{\text{ FW}} = \left[ {\left( {12.7 \times A_{{663}} } \right) - \left( {2.64 \times A_{{645}} } \right)\left( {V/\left( {1000 \times W} \right)} \right)} \right]$$

$${\mu\text{Chl }}a/g{\text{ FW}} = \left[ {\left( {22.9 \times A_{645} } \right) - \left( {2.64 \times A_{663} } \right)\left( {V/\left( {1000 \times W} \right)} \right)} \right]$$

$$\mu {\text{g }}\beta - {\text{Carotenoids}}/g{\text{ FW}} = \left[ {\left( {4.6 \times A_{{470}} } \right) - 0.268\left( {Cl.a + b} \right)} \right]\left( {V/\left( {1000 \times W} \right)} \right)$$

where: *V* = the final volume of the extract; *W* = weight of the sample.

Proline continent was measured according to the method described by Troll and Lindsley (1955) and modified by Benlaribi et al. (1990), using a UV-Spectrophotometer (SPAD-502Plus) at wavelength 528 nm. Proline content was calculated using the following equation:

$$Y = \frac{{0.62 {\text{OD}} \left( {528} \right)}}{{{\text{DW}}}}$$

*Y*: proline content μmol/g FW; OD: optical density; DW: dry weight (g).

#### Soil EC and pH measurements

The soil’s pH and EC were measured at the end of the pot experiment, in soil solution 1:2 (v/v) according to the method described by Page (1983) using an EC meter (Portable High Range EC/TDS Meter-HI99301—Hanna Instruments- USA) and a pH meter (Jenway 3505 pH/mV/Temperature Meter).

## Statistical analysis

The statistical analyses were conducted using the SAS program (SAS Institute 2009). The LSD test was used to compare between means for statistically significant differences (*p* ≤ 0.05). The effects of *Pisolithus* strain and salt stress on seedlings of *Eucalyptus globulus* were investigated using a two-way ANOVA.

## Results

### Morphological and microscopic characteristics of the collected sporocarps

**Fig. 1:**
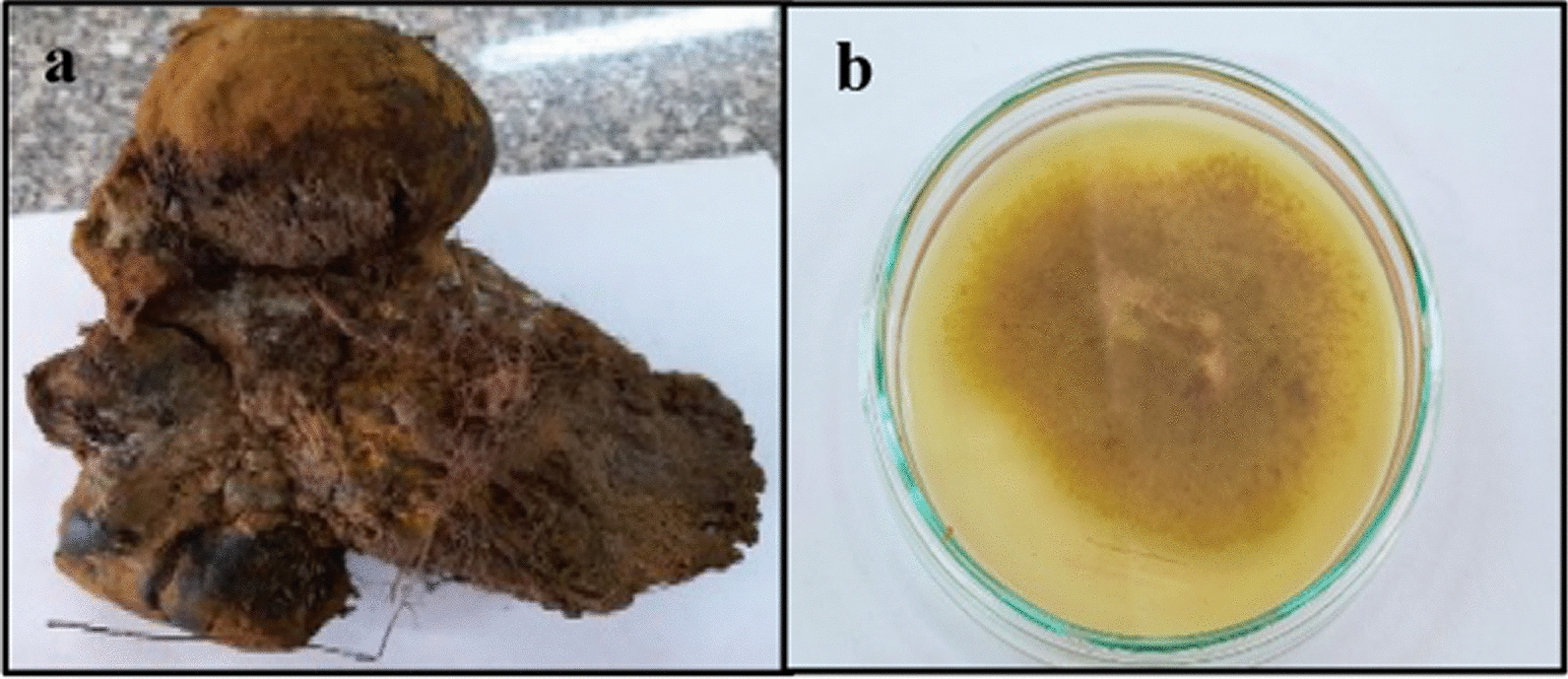
**a** Field photographs of the collected sporocarp, **b** Colony features of the isolated fungi on MMN agar medium

The morphological and microscopic features of the freshly collected sporocarps are presented in (Table S1). The preliminary identification of the sporocarps was *Pisolithus* sp. (Fig. 1a).

### Isolation of ectomycorrhizal fungus

During the incubation phase of the initial fungal development, the Petri dishes containing modified Melin-Norkrans (MMN) agar medium and inoculated with small pieces of tissue from sporocarps were examined under a stereomicroscope every day. After 20 days, the fungal isolate started to proliferate and produce visible mycelium from the tissue. The newly isolated fungus was subcultured for purification. The pure colony displayed a brownish color and cottony fungal growth (Fig. 1b).

### Molecular identification of the ectomycorrhizal isolate

The genetic analysis of pure cultures through PCR techniques generated restriction fragment length polymorphism (RFLP) fragments of varying lengths, which were submitted to GenBank for identification. The taxonomic similarities were allotted to test species based on BLAST sequence similarity analysis (https://blast.ncbi.nlm.nih.gov/Blast.cgi) including the numerous most closely matched sequences. The strain was identified as *Pisolithus tinctorius* with a similarity of 99.31% and deposited in the GenBank database with an accession number of OM125275 (https://www.ncbi.nlm.nih.gov/nuccore/OM125275). The fungal strain was preserved in the Microbial Inoculants Center, a branch of the Egyptian Microbial Culture Collection Network, under code number: EMCCN 11106. Based on the multi-alignment plots of the ITS region, (Fig. S1) displays the phylogenetic trees of the identified strain.

### Aseptic synthesis of ectomycorrhizae (In vitro mycorrhization)

**Fig. 2:**
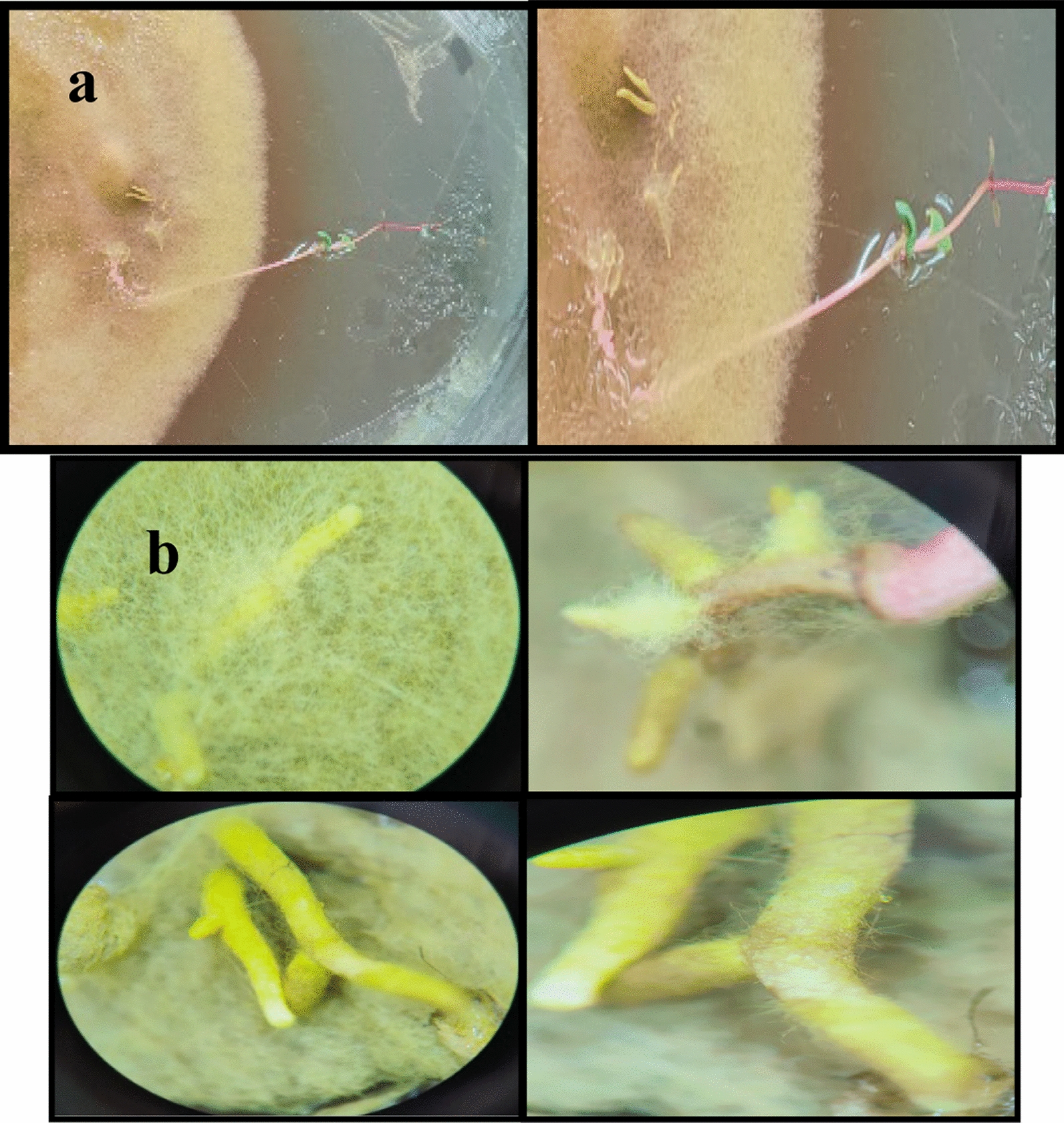
**a** In vitro ectomycorrhizal synthesis between *Pisolithus* *tinctorius* and *Eucalyptus* sp. in sterile MMN medium, **b** Microscopic examination of *Eucalyptus* sp. roots inoculated with *Pisolithus* *tinctorius*

The germinated *Eucalyptus* sp. seeds were inoculated with the fungal isolate *Pisolithus* sp. in Petri dishes to test its capability to form ectomycorrhizae. The mycorrhizal status and mycorrhizal evolution of the roots were observed at regular intervals (every three days), which revealed seedlings’ development (Fig. 2a) during 30 days of the in-vitro mycorrhization. Microscopic examination of the plant roots (Fig. 2b) showed the mantel and external mycelium.

The tested *Pisolithus* strain apparently formed a typical ectomycorrhizal structure with *Eucalyptus* sp. (Fig. 2b). The typical ectomycorrhizal formation revealed mycelial spreading (Fig. 2b) with well-developed emanating hyphae (external mycelium). The short ectomycorrhizal roots were completely enveloped with cottony hyphae forming the mantle (Fig. 2b).

Microscopic examinations of the root system showed that most lateral roots were in contact with the growing colony and were completely enveloped by the fungal hyphae (Fig. 2b). The mantle color of the mycorrhizae was similar to that of the pure culture of *Pisolithus* sp.

**Fig. 3:**
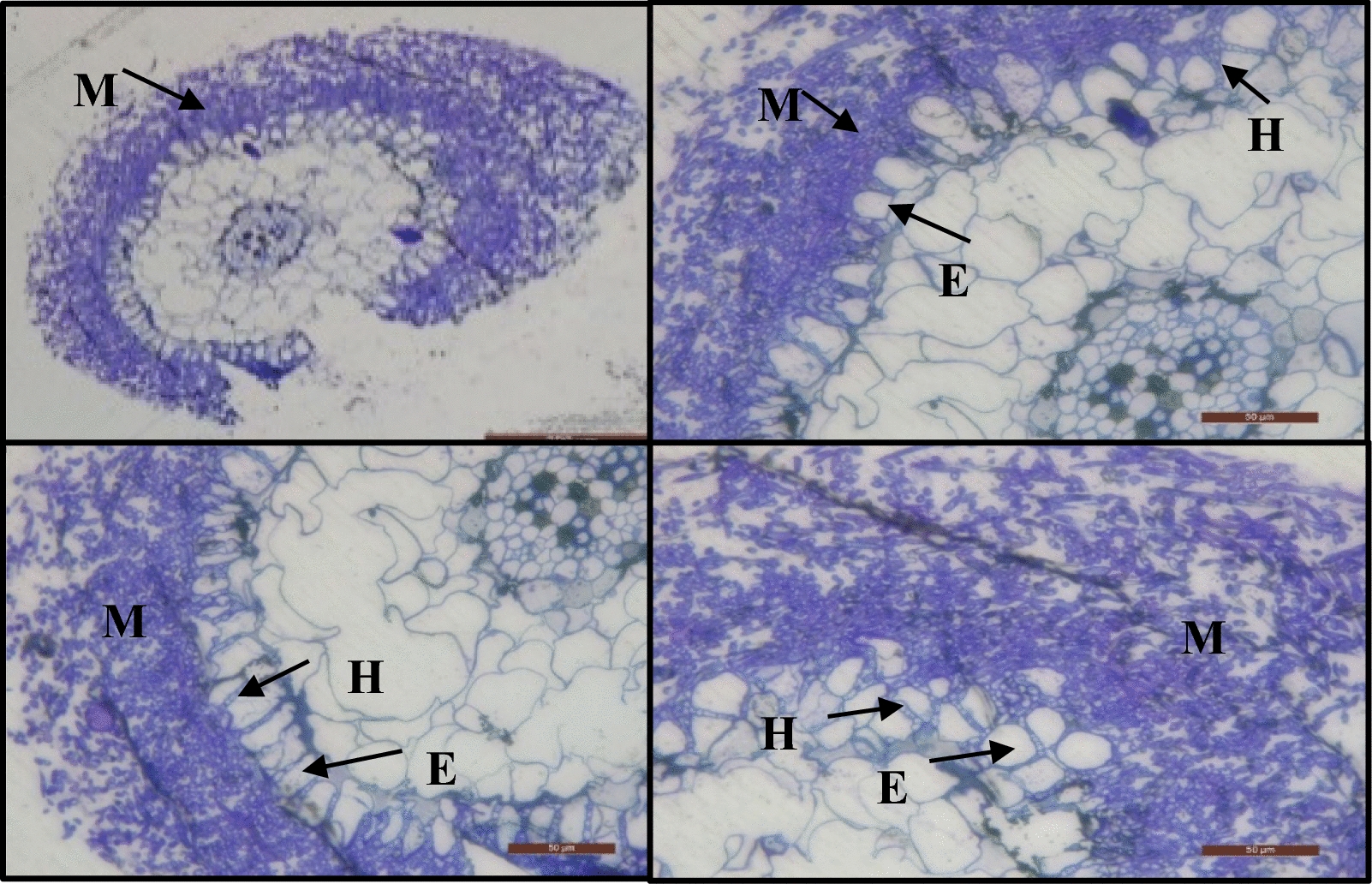
Semithin plastic sections on the ectomycorrhizal association between the roots of *Eucalyptus* sp. and *Pisolithus* sp.; Mantle layers around the root (M); the Hartig net (H) appears between the epidermal cells (E)

Semithin light microscopy analysis of *Eucalyptus* sp. seedling roots revealed a multilayered mantle with well-stained compact hyphae in the inner layers and loose, lightly stained external mycelia in the outer layers (Fig. 3).

### The growth performance of *Pisolithus tinctorius* at different pH levels

**Fig. 4:**
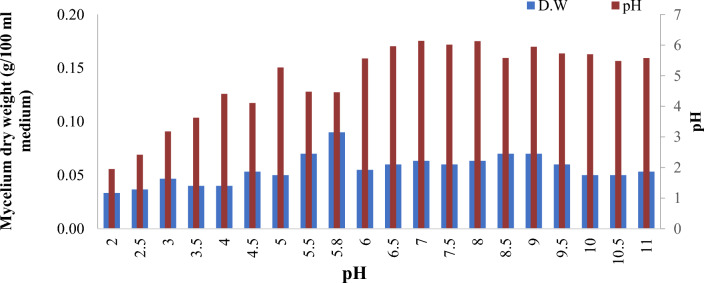
The mycelium dry weight (g) of *Pisolithus* *tinctorius* in different pH levels (2 to 11 ±  0.5) incubated at 28 °C for 24 days and the final pH of each treatment

*Pisolithus tinctorius* strain was subjected to different pH levels ranging from 2 to 11. The pH of the medium was measured at the end of the incubation period, as shown in Fig. 4. The optimal pH range for the *Pisolithus tinctorius*’ growth was found to be between pH 4.5 and 11. While the highest mycelium dry weight was observed at pH 5.8 of 0.09 g/100 ml medium. The final pH of the medium at the end of the 24-day incubation period showed the strain’s capability to produce acidic substances that reduced the pH of the medium.

### The growth performance of *Pisolithus tinctorius* in different temperatures

**Fig. 5:**
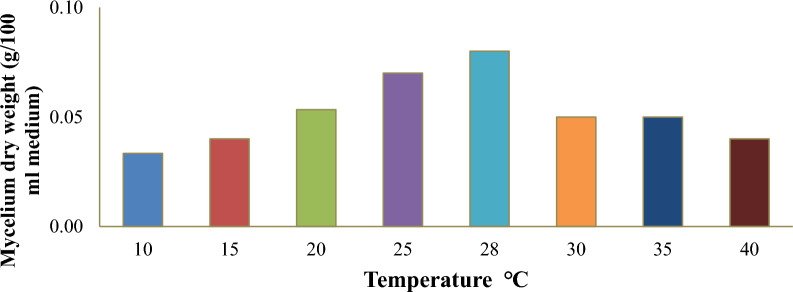
The mycelium dry weight (g) of *Pisolithus* *tinctorius* in different temperatures (15–30 °C) incubated for 24 days

Data illustrated in Fig. 5 reveal the mycelium dry weight of the tested strain grown at different temperatures. The optimum temperature for the growth of *Pisolithus tinctorius* ranged between 20 and 28 °C. The *Pisolithus tinctorius* recorded the highest mycelium dry weight at 28 °C of 0.08 g/ 100 ml medium which was deemed as the optimum temperature.

### The growth performance of *Pisolithus tinctorius* NaCl concentrations

**Fig. 6:**
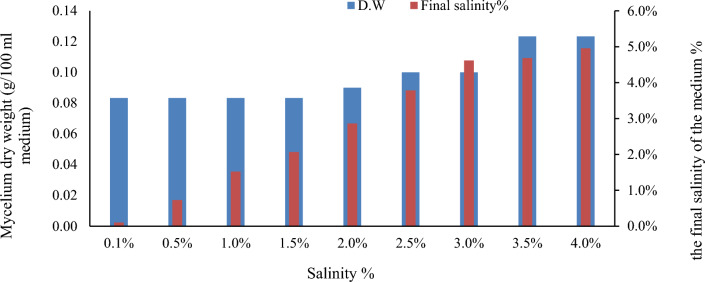
The mycelium dry weight (g) of Pisolithus tinctorius in different NaCl concentrations (0.1–4.0%) incubated at 28 °C for 24 days and the final EC of each treatment

*Pisolithus tinctorius* demonstrated clear growth variations at different NaCl concentrations, as illustrated in Fig. 6. Overall, the biomass of *Pisolithus tinctorius* was not significantly impacted by increasing NaCl levels. Across all NaCl concentrations, the mycelial dry weight exhibited an increase compared to the standard medium (control). *Pisolithus tinctorius* displayed the highest mycelial dry weight growth at a 4% NaCl concentration of 0.12 g/100 ml medium.

### The growth performance of *Eucalyptus globulus* cultivated on sandy soil as affected by the salt concentration in the irrigation water and *Pisolithus tinctorius* inoculation

The growth parameters recorded for *Eucalyptus globulus* grown in sandy soil and subjected to different treatments of salty water and inoculation with *Pisolithus tinctorius* presented clear variations (Fig.  S2).

Generally*, Eucalyptus globulus* seedlings inoculated with *Pisolithus tinctorius* showed notable improvements in various parameters when compared to non-mycorrhizal treatments. These enhancements included a significant increase in shoot height (48.468 cm), root length (42.166 cm), root fresh weight (1.978 g), root dry weight (0.614 g), and proline content (34.137 µmol/g fresh weight). In contrast, the control group exhibited a significant rise in chlorophyll (a, b) and carotenoid levels of 0.029, 0.021, and 0.012 µg/g fresh weight, respectively (Table 1).

**Table 1:** Effect of salted irrigation water and *Pisolithus tinctorius* on the growth parameters and some biochemical constituents of *Eucalyptus globulus* in sandy soil

Treatments		Parameters									
		Shoot height (cm)	Root length (cm)	Shoot fresh weight (g/plant)	Root fresh weight (g/plant)	Shoot dry weight (g/plant)	Root dry weight (g/plant)	Chlorophyll a (µg/g FW)	Chlorophyll b (µg/g FW)	Carotenoids (µg/g FW)	Proline (µmol/g FW)
Hoogland (0.6 dS/m)	Control	47.370 ± 0.470	44.830 ± 0.441	4.850 ± 0.441	1.640 ± 0.441	1.940 ± 0.441	0.250 ± 0.441	0.049 ± 0.044	0.038 ± 0.044	0.023 ± 0.044	28.526 ± 0.441
	*Pisolithus tinctorius*	53.500 ± 10.970	36.000 ± 0.577	5.910 ± 2.651	1.160 ± 0.497	1.857 ± 0.615	0.497 ± 0.199	0.039 ± 0.004	0.026 ± 0.004	0.020 ± 0.004	15.186 ± 0.440
	Mean	50.435	40.415	5.380	1.400	1.898	0.373	0.044	0.032	0.022	21.856
6 (dS/m)	Control	45.500 ± 15.782	28.667 ± 8.876	5.720 ± 3.098	1.030 ± 0.631	1.990 ± 1.112	0.440 ± 0.286	0.019 ± 0.006	0.016 ± 0.001	0.008 ± 0.002	6.284 ± 1.941
	*Pisolithus tinctorius*	55.670 ± 7.219	42.330 ± 3.371	6.440 ± 1.763	1.840 ± 0.682	2.370 ± 0.542	0.810 ± 0.240	0.022 ± 0.003	0.013 ± 0.001	0.009 ± 0.001	28.959 ± 3.702
	Mean	50.585	35.499	6.080	1.435	2.180	0.625	0.020	0.015	0.009	17.622
8 (dS/m)	Control	20.830 ± 3.444	37.830 ± 3.167	1.220 ± 0.485	0.360 ± 0.119	0.397 ± 0.139	0.270 ± 0.087	0.027 ± 0.002	0.018 ± 0.001	0.009 ± 0.001	8.402 ± 0.350
	*Pisolithus tinctorius*	43.870 ± 5.962	48.333 ± 3.844	3.780 ± 0.457	1.840 ± 0.362	1.497 ± 0.317	0.530 ± 0.089	0.007 ± 0.001	0.011 ± 0.001	0.004 ± 0	24.480 ± 1.545
	Mean	32.350	43.082	2.500	1.100	0.947	0.400	0.017	0.015	0.007	16.441
10 (dS/m)	Control	22.800 ± 3.650	27.830 ± 1.202	0.950 ± 0.254	0.560 ± 0.049	0.270 ± 0.035	0.220 ± 0.020	0.019 ± 0.001	0.011 ± 0	0.008 ± 0.001	21.907 ± 1.071
	*Pisolithus tinctorius*	40.830 ± 3.167	42.000 ± 3.686	3.900 ± 0.514	3.073 ± 0.322	1.230 ± 0.062	0.620 ± 0.107	0.009 ± 0	0.007 ± 0	0.008 ± 0	67.921 ± 1.414
	Mean	31.815	34.915	2.425	1.817	0.750	0.420	0.014	0.009	0.008	44.914
Mean	Control	34.125	34.789	3.185	0.898	1.149	0.295	0.029	0.021	0.012	16.280
	*Pisolithus tinctorius*	48.468	42.166	5.008	1.978	1.738	0.614	0.019	0.014	0.010	34.137
LSD 5%	Salt concentrations	16.633	8.562	3.411	0.935	1.119	0.341	0.007	0.005	0.005	3.635
	ECM strain	11.762	6.054	2.412	0.661	0.791	0.241	0.005	0.004	0.004	2.570
	Salt × ECM	23.523	12.109	4.823	1.322	1.582	0.482	0.010	0.007	0.007	5.140

Values are the averages of 3 replicates ± SE (standard error). The difference among means was considered significant according to t Tests (LSD) (*p* < 0.05)

Regarding the effect of salt concentration (EC) in irrigation water on the growth parameters and biochemical constituents of *Eucalyptus globulus*, it is clear that most parameters were higher in plants irrigated with 6 dS/m saline water, even though there was no significant difference between *Eucalyptus globulus* seedlings irrigated with Hoogland (0.6 dS/m) and those irrigated with 6 dS/m water.

All the measured parameters showed a significant decrease upon increasing the EC of irrigation water to 8 and 10 dS/m, with the exception of proline. Notably, proline levels in *Eucalyptus globulus* seedlings watered with 10 dS/m significantly increased to 44.914 µmol/g fresh weight when compared to those irrigated with Hoogland, 6, and 8 dS/m.

Considering the combined effect of the irrigation water’s EC and the inoculation of seedlings with *Pisolithus tinctorius*, it is clear that *Eucalyptus globulus* seedlings inoculated by *Pisolithus tinctorius* showed an improvement in all growth parameters compared to the non-inoculated seedlings. The greatest shoot height, root length, shoot fresh weight, root fresh weight, shoot dry weight, and root dry weight were observed in seedlings inoculated with *Pisolithus tinctorius* and irrigated with 6 dS/m saline water of 55.670 cm, 42.33 cm, 6.44, 1.84, 2.37, and 0.810 g/plant, respectively. The highest significant proline content was found in seedlings irrigated with 10 dS/m water and inoculated with *Pisolithus tinctorius* at 67.921 µmol/g fresh weight.

The biochemical data shows a notable reduction in chlorophyll (a, b) and carotenoids contents in seedlings inoculated with *Pisolithus tinctorius* compared to uninoculated seedlings (control) across all treatments. Additionally, inoculated *Eucalyptus globulus* seedlings displayed a significant decrease in proline content of 15.186 µmol/g fresh weight when irrigated with Hoogland (0.6 dS/m), and a significant increase to 28.959, 24.48, and 67.921 µmol/g fresh weight when irrigated with water of 6, 8, and 10 dS/m, respectively, compared to the control in each treatment.

### NPK contents in *Eucalyptus globulus* cultivated on sandy soil as affected by the salt concentration in the irrigation water and *Pisolithus tinctorius* inoculation.

**Fig. 7:**
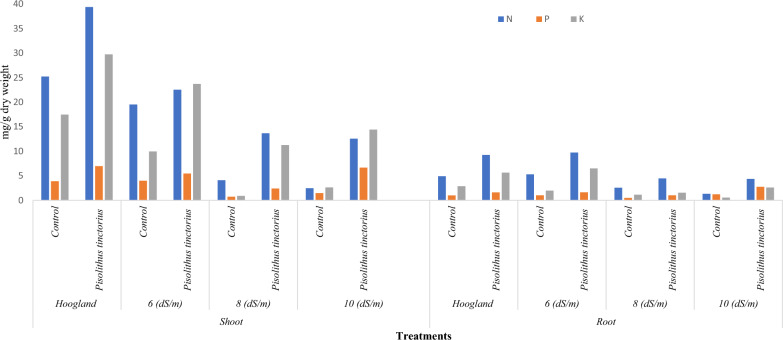
NPK contents of *Eucalyptus* *globulus* cultivated in sandy soil as affected by salted irrigation water (6, 8, and 10 dSm^-1^) and *Pisolithus* *tinctorius* strain

The data in Fig. 7 shows the evident impact of *Pisolithus tinctorius* on the NPK contents in *Eucalyptus globulus* seedlings irrigated with salted water compared to non-inoculated seedlings.

In general, all treatments that were inoculated with *Pisolithus tinctorius* generally displayed greater NPK levels in shoots and roots compared to non-inoculated treatments.

The highest NPK concentrations were found in the shoot (39.37, 6.95, and 29.72 mg/g dry weight) and the root (9.24, 1.6, and 5.63 mg/g dry weight) of *Eucalyptus* seedlings inoculated with *Pisolithus tinctorius* and irrigated with Hoogland (0.6 dS/m). Furthermore, it was noticed that *Eucalyptus globulus* seedlings’ NPK content decreased with increased salt concentrations in irrigation water in all treatments.

#### The pH and EC (dS/m) of sandy soil cultivated by *Eucalyptus globulus* and treated with different salt irrigation water and *Pisolithus tinctorius* inoculation.

**Fig. 8:**
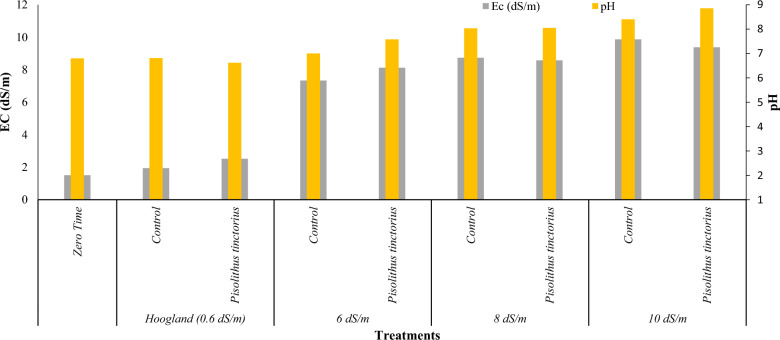
pH and EC (dS/m) of the sandy soil at the end of the pot experiment

The data presented in Fig. 8 indicates the changes in electrical conductivity (EC) and pH levels of sandy soil at the end of the experiment. Across all treatments, there was a general increase in both EC and pH levels when compared to the initial measurement (zero-time). These findings suggest that raising the salt content in the irrigation water raised the pH and EC values in all treatments.

The soil treated with *Pisolithus tinctorius* and irrigated with Hoogland at 0.6 dS/m and 6 dS/m had the highest electrical conductivity (EC) compared to the control soil (soil without treatment) with values of 2.52 and 8.12 dS/m for the two levels of irrigation, respectively. The treatments irrigated with 8 and 10 dS/m showed the highest EC in the control soil with values of 8.74, and 9.87, and the lowest EC in the soil inoculated with *Pisolithus tinctorius*, with values of 8.58, and 9.38 dS/m, respectively.

At the end of the pot experiment, the soil pH levels revealed a slight difference between the sub-treatments that were irrigated using the same concentration of salt water. Additionally, it was noticed that the pH levels of the soil increased as the salt concentration in the irrigation water increased. Initially, the soil had a pH level of 6.8, and it reached its maximum pH value of 8.85 at the end of the treatment when irrigated with 10 dS/m water.

## Discussions

### Isolation and identification of ectomycorrhizal fungus

Despite recent advances in molecular methods for identifying ectomycorrhizal fungi, classical methodologies for identifying ECM fungi offer several advantages. Therefore, tracking sporocarps’ characteristics remains the most reliable way to assess and identify ECM’s fungal genus. However, molecular taxonomy is well supported for identifying fungal species, compared with morphoanatomical-based taxonomy. Therefore, this investigation combined the studies of morphoanatomical and molecular characterization of ECM fungi, followed by phylogenetic characterization to ensure accurate identification of the ectomycorrhizal fungi (Mrak et al. 2017; Kumar and Atri 2018).

According to Brundrett et al. (1996); Agerer and Rambold (2004) the collected sporocarp was identified as *Pisolithus* sp.

The pure colony displayed a brownish color and cottony fungal growth. The ectomycorrhizal isolate was corroborated as *Pisolithus* sp. according to the results of Brundrett et al. (1996); Obase et al. (2009); Repáč (2011).

The genetic analysis of the *Pisolithus* isolate’s pure cultures using 18S rDNA and PCR was subjected to the GenBank. The tested ectomycorrhizal fungus was identified as *Pisolithus tinctorius* with a similarity of 99.31% and an accession number of OM125275.

### Aseptic synthesis of ectomycorrhizae (In vitro mycorrhization)

The morphological and microscopic characteristics of the roots obtained by the in-vitro mycorrhization are in line with those reported by Martins (2008); Sehgal and Sagar (2022) who mentioned that mycorrhizas obtained by in-vitro synthesis forms mantles and Hartig nets with comparable structures, and the mantle thickness and the number of hyphae penetrating between cortical cells may vary with the substrate, the synthesis method used, the fungal genus and the plant genus.

These preliminary results from the semithin light microscopy indicate that *Pisolithus* sp. was able to colonize the roots of *Eucalyptus* seedlings at the end of the experiment, once the hyphae had contacted the root surface (Sehgal and Sagar 2022). These results are consistent with those reported by Misbahuzzaman and Ingleby (2005); Sharma et al. (2009) who stated that *P. tinctorius* has the ability to form a mantle that differentiates to loosen hyphae that form the outer mantle and gradually becomes compacted in the inner. While Misbahuzzaman and Ingleby (2005) noted a dual-layered thickened mantle in their examination of *P. tinctorius.*

### The growth performance of *Pisolithus tinctorius* at different pH levels

Maintaining the appropriate pH level is a crucial factor for the growth of ectomycorrhizal fungi. The pH level impacts the microbial activity by affecting the availability of nutrients and the ionization of inorganic and organic constituents in the solutions. It also plays a significant role in the enzymatic activity of the mycelium (Voroney 2007). Different researchers have shown that ECM fungi prefer acidic conditions with a pH range of 4.5–6.0 for optimal growth and the mycelial growth decreases at pH levels below 4.5. However, Krishna Sundari and Adholeya (2003) reported that, members of the Agaricales (except *Laccaria laccata*) and Aphyllophorales orders prefer a neutral pH. Whereas members of the Sclerodermatales order prefer acidic pH. In a recent study, *Pisolithus tinctoriu*s demonstrated varying behavior at different pH levels and demonstrated the ability to thrive in alkaline conditions and convert them to acidic by the end of the incubation period. Many researchers have found that during the development of the fungal mycelium, it produces a series of organic acids which consequently lower the pH level of the growth medium (Suárez et al. 2023). Therefore, measuring the pH level of the medium at the end of the experiment was one of our main objectives to identify the capability of the tested strains to function as biological buffers in reducing the acidic and/or alkaline pH of the mycelium growth medium.

### The growth performance of *Pisolithus tinctorius* in different temperatures

The growth performance of *Pisolithus tinctorius* in different temperatures is inconsistent with those of Deshaware et al. (2021), who reported that 25 °C was the ideal temperature for the mycelial growth of the majority of macrofungal species. In this study, the mycelium growth of *Pisolithus tinctorius* drastically decreased with decreasing and increasing temperatures over the optimal temperature range of 20–28 °C. The reduction of mycelial growth below the optimal temperature can be traced to the fungus’s reduced metabolic activities, which reduce the absorption of essential nutrients needed for growth. Whereas the reduction of mycelial growth above the optimal temperature may be traced to the denaturation of important enzymes that catalyze fungal metabolic processes, which indirectly affect mycelium growth (Peng et al. 2019).

### The growth performance of *Pisolithus tinctorius* NaCl concentrations

*Pisolithus tinctorius*’s mycelial dry weight increased at all NaCl concentrations compared to the control. This finding is consistent with Li et al. (2022) on *Pisolithus tinctorius.*

The growth of *Pisolithus tinctorius* at various NaCl concentrations is consistent with those presented by Li et al. (2022), who indicated that *Pisolithus tinctorius* is a moderately NaCl-tolerant species. In the same consequence, Thiem et al. (2020) stated that ECM fungal isolates could be considered salinity-tolerant if they maintained more than 50% of their mycelial weights under specific saline conditions compared with non-saline conditions. Therefore, it can be inferred that this strain (*Pisolithus tinctorius*) is halotolerant. Moreover, *Pisolithus tinctorius* recorded increases in the final salinity of the medium’s supernatant at the end of the experiment (24-day incubation period) compared with the initial salinity. Increasing the salinity in the medium at the end of the incubation period could be attributed to decreases in the suspension volume of the medium due to evaporation during the incubation period and the production of different metabolites by the fungi during the growth. and could indicate the ability of this strain to withstand high salinity (up to 5%) for an extended period.

### The growth performance of *Eucalyptus globulus* cultivated on sandy soil as affected by the salt concentration in the irrigation water and *Pisolithus tinctorius* inoculation

Salt stress negatively affects plants in various ways. It triggers complex physiological and biochemical reactions, including the closure of stomata, osmotic stress, ion toxicity, reduced transpiration, reduced water uptake by roots, and oxidation–reduction reactions (Selim and Zayed 2017). These reactions have the potential to damage chloroplasts in leaves, disrupt various metabolic processes, and disrupt the plant’s water and nutrient balance (Gong et al. 2011; Guerrero-Galán et al. 2019; Bai et al. 2021a, b; Balasubramaniam et al. 2023). Consequently, a plant’s ability to thrive in high-salinity soil largely depends on its capacity to overcome these challenges (Balasubramaniam et al. 2023; Rose et al. 2024).

Our results indicate a significant improvement in the growth and tolerance of seedlings inoculated with ectomycorrhizal (ECM) fungi compared to those that were not inoculated (non-ectomycorrhizal). This enhancement can be attributed to various mechanisms, including those described by Smith and Read (2008) and Guerrero-Galán et al. (2019), that ECM fungi improve plants’ tolerance to salt stress by enhancing soil structure through fungal mycelial networks which facilitate better plant rooting, resulting in greater growth for mycorrhizal plants compared to non-mycorrhizal ones (Rillig and Mummey 2006).

Additionally, ECM fungi impact several processes within host plant tissues, such as the redistribution of sodium ions (Na^+^) and extrude Na^+^ from plant cells, particularly in the shoots, to prevent or reduce its accumulation in photosynthetic tissues (Calvo-Polanco et al. 2008a, 2008b; Li et al. 2012; Guerrero-Galán et al. 2019). This strategy involves storing Na^+^ in the root apoplasm instead of in the vacuoles of root cells, thereby avoiding the entry of Na^+^ into the symplastic pathway and protecting the plant’s photosynthetic apparatus from sodium toxicity (Ottow et al. 2005; Guerrero-Galán et al. 2019).

While Bradshaw (2000) also stated that ectomycorrhizal fungi can help mitigate the toxicity of NaCl and other specific ions by regulating ion absorption into the plant roots by acting as a filter or barrier between the external environment and the root, which prevents harmful Na^+^ ions from reaching the root cortex (Guerrero-Galán et al. 2019).

Likewise, inoculating seedlings with ectomycorrhizal fungi (ECM) improves water balance by enhancing water absorption (Bai et al. 2021a, b) and regulating water transport under salt stress (Guerrero-Galán et al. 2019; Llanes et al. 2021; Rose et al. 2024). Also, ECM can mitigate the inhibition of aquaporins, which facilitate water transport in roots under various soil stress conditions, by modifying the expression and abundance of plant aquaporins (Xu et al. 2015; Peter et al. 2016; Guerrero-Galán et al. 2019). These results are consistent with our findings, where all seedlings inoculated with *Pisolithus tinctorius* showed higher shoot and root fresh weights under salt stress compared to uninoculated seedlings.

The biochemical results in *Eucalyptus globulus* seedlings can be attributed to *Pisolithus tinctorius*’*s* ability to regulate biochemical components under osmotic salt stress. Proline serves as the primary organic osmoregulatory in shoots, offering osmo-protective functions and acting as an endogenous regulator that helps plants withstand salt stress. Our study indicates that inoculating *Eucalyptus* sp. seedlings with *Pisolithus tinctorius* in saline conditions enhances growth and promotes proline accumulation in plant tissues. This accumulation is a known adaptation to salt stress, as noted by Thiem et al. (2020) and Usman et al. (2021). Conversely, Bradshaw (2000) reported that inoculating seedlings with ECM significantly reduced proline content, suggesting that such inoculation may lessen proline’s necessity as an osmoregulatory agent.

Previous research has shown that under salt stress conditions, plants’ chloroplasts tend to disintegrate, and the synthesis of chlorophylls and carotenoids is inhibited. However, studies have also found that ectomycorrhizal fungi can increase the chlorophyll content of seedlings inoculated by *P. thunbergii* under such conditions. In our study, we observed that the chlorophyll content was lower in mycorrhizal seedlings compared to control seedlings under most NaCl treatments. The decrease in chlorophyll content in mycorrhizal plants is a complex phenomenon that can be influenced by various biological and environmental factors. For instance, the amount of pigments in mycorrhizal plants can be reduced under the influence of salinity stress due to the ability of mycorrhizal fungi to facilitate a beneficial symbiotic interaction with seedlings, which in turn enhances some physiological processes in plants like photosynthetic efficiency Ye et al. (2019); Baltazar-Bernal et al. (2022). Additionally, ectomycorrhizal fungi have the capability to change the pathway of nitrogen metabolism in certain physiological processes, such as chlorophyll and carotenoid production, in exchange for the production of some compounds, such as proline (Nasir Khan et al. 2010). Mycorrhizal fungi boost nutrient absorption in plants, which may lead to decreased chlorophyll production when less sunlight is needed for photosynthesis.

### NPK contents in *Eucalyptus globulus* cultivated on sandy soil as affected by the salt concentration in the irrigation water and *Pisolithus tinctorius* inoculation.

NPK levels in *Eucalyptus globulus* grown on sandy soil are influenced by salt concentrations in irrigation water and *Pisolithus tinctorius* inoculation. The results revealed an increase in the nutrient levels in seedlings inoculated with ECM compared to those uninoculated. These findings are consistent with the prior research of Guerrero-Galán et al. (2019); Usman et al. (2021), who suggested that ECM are able to keep the seedlings in a healthier state by improving mineral nutrition and water absorption of the plants to through provides exchangeable surfaces to facilitate these processes (Bradshaw 2000). ECM also boosts K^+^ levels in host leaves, especially under K^+^-deficient conditions, which leads to a higher K/Na ratio in ECM plants than in non-ECM plants as evidenced in the studies of Luo et al. (2009); Garcia et al. (2014); Guerrero-Galán et al. (2019); Kulczyk-Skrzeszewska and Kieliszewska-Rokicka (2022). Additionally, EMF can elevate Ca^2+^ (Ma et al. 2014; Guerrero-Galán et al. 2019), phosphorus levels (Guerrero-Galán et al. 2019) and nitrogen levels in ectomycorrhizal seedlings (Sa et al. 2019; Guerrero-Galán et al. 2019). In the same consequence, Luo et al. (2011) demonstrated that ectomycorrhizal poplar (*P. involutus*) maintained a better nutritional status, resulting in reduced stress responses and enhanced photosynthetic activity. These findings align with our results, which indicated that plants inoculated with *Pitholithus* sp. exhibited higher NPK levels compared to uninoculated seedlings.

### The pH and EC (dS/m) of sandy soil cultivated by *Eucalyptus globulus* and treated with different salt irrigation water and *Pisolithus tinctorius* inoculation

Our study analyzed the effects of different salt irrigation water and *Pisolithus tinctorius* inoculation on the pH and EC (dS/m) levels of sandy soil cultivated by *Eucalyptus globulus* at the end of the experiment. Our results revealed an increase in the pH level due to increasing the Ec of the irrigation water, which is consistent with Van and Thanh (2021), who reported that increased salinity causes cation exchange between Na^+^ and H^+^, which can lead to a higher H^+^ ion concentration in the soil solution, which in turn can lower the soil’s pH level, and suggested that the presence of an increase in the pH level of the soil is mostly negligible. While Sreenivas and Reddy (2008) stated an increase in pH levels with increasing salinity due to the presence of sodium bicarbonate and carbonate.

## Supplementary Information

Additional file 1.

Additional file 2.

## Data Availability

The data sets used and/or analyzed during the current study are available from the corresponding author upon reasonable request. *Pisolithus tinctorius* was deposited in GenBank at (https://www.ncbi.nlm.nih.gov/nuccore/OM125275).

## Declarations

### Human or animal participants

This article contains no studies with human participants or animals performed by any of the authors. The manuscript contains no images or other personally identifiable information about the participants that could compromise their anonymity.
