# Supplementary material for: Evaluating the effectiveness of Pisolithus tinctorius in enhancing the Eucalyptus’ resistance to salt stress
Source: AMB Express. 2025 Jan 4;15:4. doi: 10.1186/s13568-024-01799-w (PMC11700078; doi:10.1186/s13568-024-01799-w)
Supplement: Supplementary file 1 — Additional file 1. [file 13568_2024_1799_MOESM1_ESM.docx]

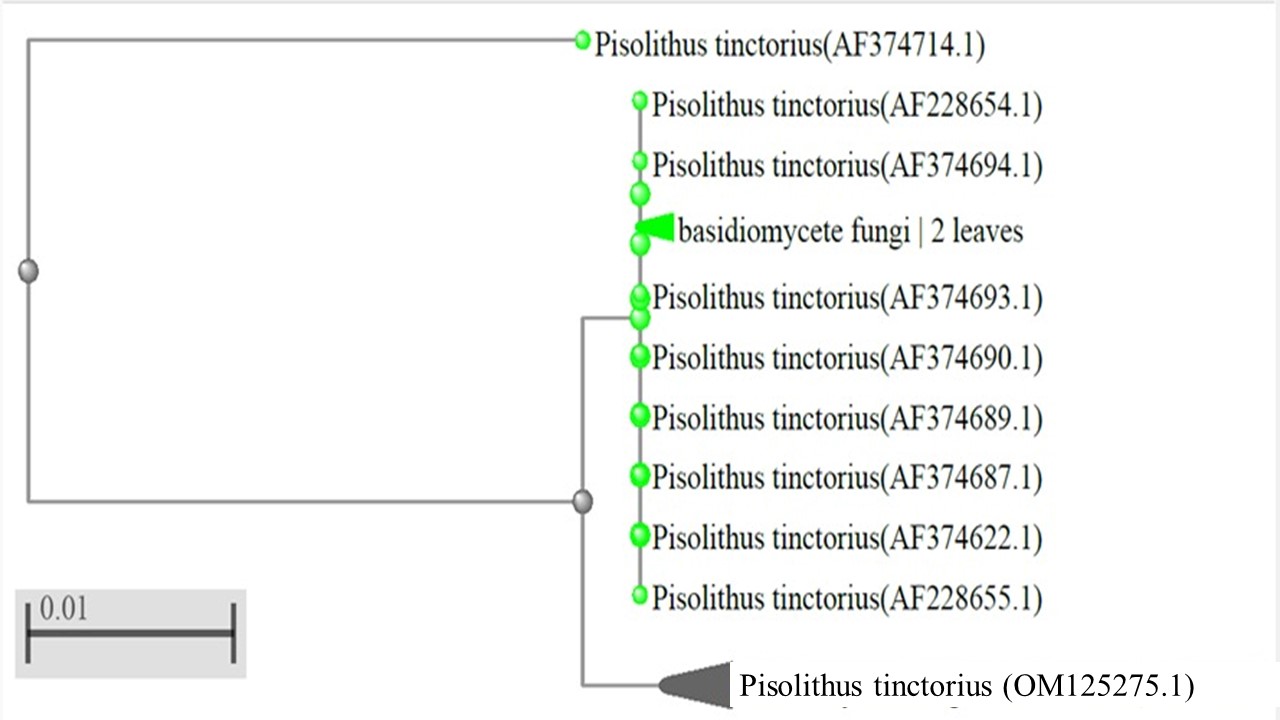


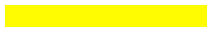


**Fig. S1** Phylogenetic tree of *Pisolithus tinctorius* based on ITS region gene sequences using specific ITS1 primers.


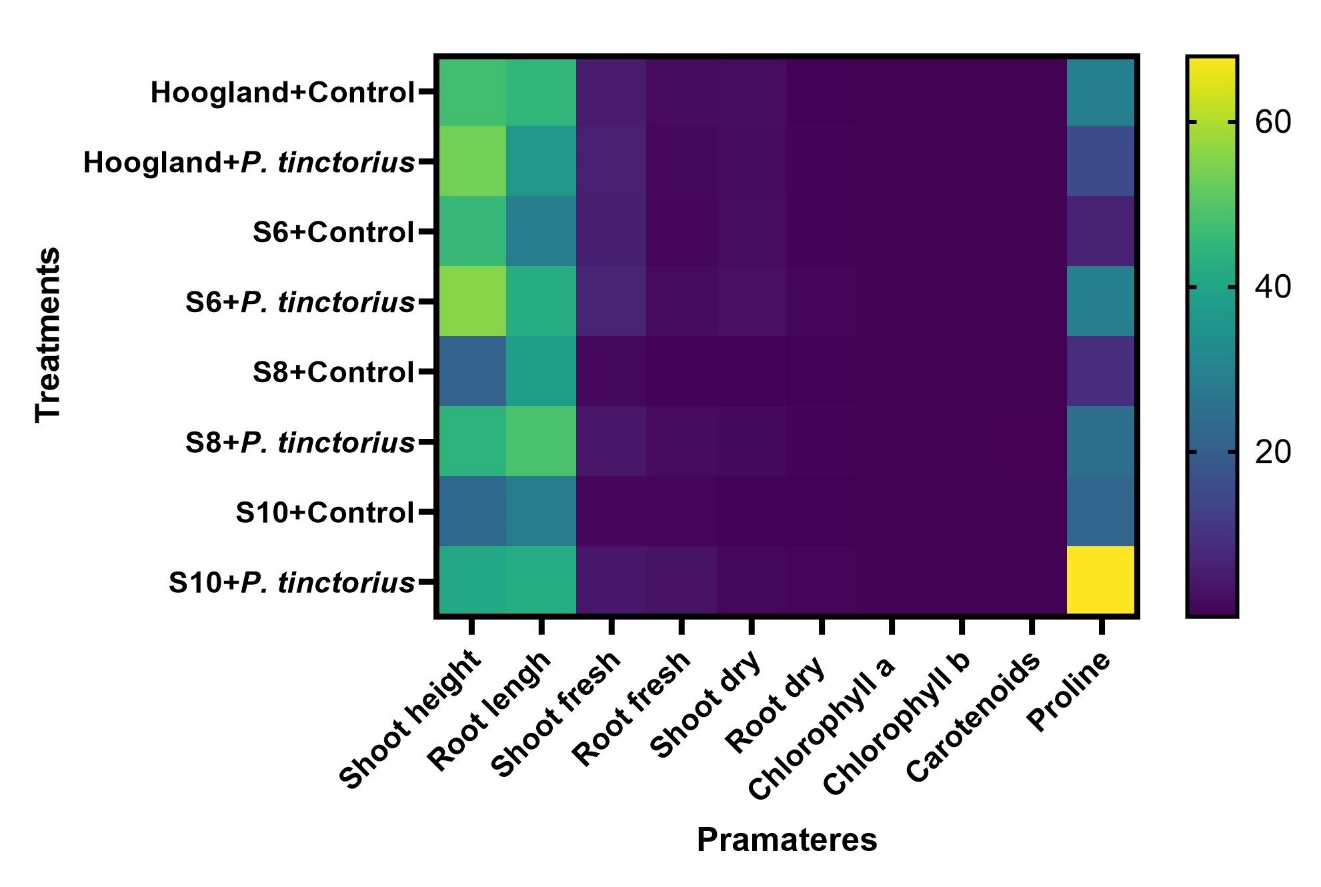


**Fig. S2** Effect of salted irrigation water and *Pisolithus tinctorius* on the growth parameters and some biochemical constituents of *Eucalyptus globulus* in sandy soil
