# Supplementary material for: Evaluating the effectiveness of Pisolithus tinctorius in enhancing the Eucalyptus’ resistance to salt stress
Source: AMB Express. 2025 Jan 4;15:4. doi: 10.1186/s13568-024-01799-w (PMC11700078; doi:10.1186/s13568-024-01799-w)
Supplement: Supplementary file 2 — Additional file 2. [file 13568_2024_1799_MOESM2_ESM.docx]

**Table S1** Morphological and microscopic features of collected *Pisolithus* sp. sporocarps.

| General characteristics of the sporocarps | |
| --- | --- |
| Host | *Eucalyptus* sp. |
| Cap shape | Ball |
| Cap surface | Smooth |
| Cap colour | Brown |
| Hymenium | Absent |
| Stem colour | Absent |
| Stem shape | Absent |
| Gill-attachment | Absent |
| Veil ring | Absent |
| Flesh colour | Brown |
| Spore print colour | Brown |
| Spore shape | Globose |
| Spore wall | Echinate |
| Suggested Genus | *Pisolithus* sp. |
